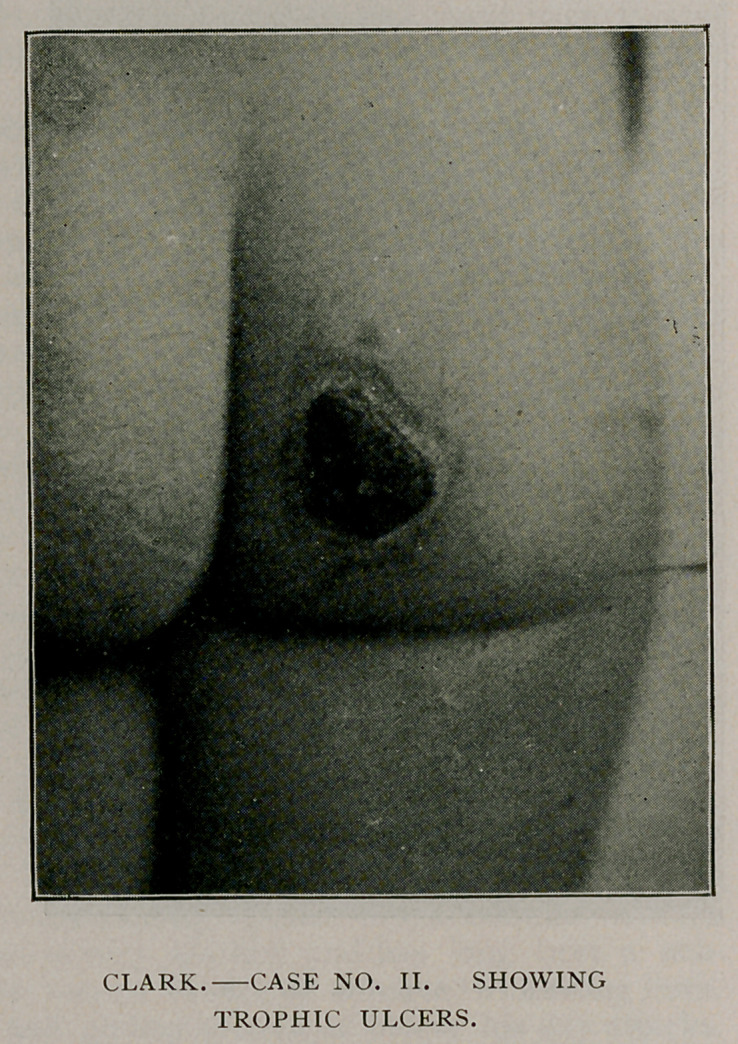# Report of Two Cases of Hysteria Major Associated with Epilepsy1Read before the Medical Society of the County of Livingston, at its semi-annual meeting at Caledonia, N. Y.

**Published:** 1899-02

**Authors:** L. Pierce Clark

**Affiliations:** Sonyea, N. Y., First assistant physician at Craig Colony; Fellow of New York Academy of Medicine and New York Neurological Society, etc.


### REPORT OF TWO CASES OF HYSTERIA MAJOR ASSOCIATED WITH EPILEPSY.[1]

BY L. PIERCE CLARK, M. D., Sonyea, N. Y.,
First assistant physician at Craig Colony; Fellow of New York Academy of Medicine and New York Neurological Society, etc.

THE differential diagnosis of many allied diseases is very difficult, especially in the so-called functional neuroses. We all know this statement is particularly applicable to the differential diagnosis of an anomalous epilepsy from an anomalous hysteria. We frequently see at various times nearly if not quite all of the pathognomonic symptoms of hysteria tangled with the symptoms of epilepsy in such a manner that it is almost impossible to say that this or that symptom is hysterical or epileptic.

It is a well-known fact among epileptologists that isolated hysterical symptoms accompany epileptic fits, and still epilepsy in such cases never loses its dignity as a separate and distinct disease. It is not hystero-epilepsy. This latter disease is no longer classed with the epilepsies, but with hysteria, where it rightly belongs.

It is uncommon to find hysteria major associated with grand mal epilepsy in the same case and at the same time have the attacks of the one alternate with the other at fairly regular intervals, or to have one follow immediately after the other.

---

1. Read before the Medical Society of the County of Livingston, at its semi-annual meeting at Caledonia, N. Y.

But two cases of hysteria major associated with epilepsy have occurred at the Craig Colony in over 450 admissions. I therefore take great pleasure in presenting them for study.

CASE I.—C. D., male, aged 24, single, no occupation. Has had epilepsy since twelve years of age. The cause was attributed to an attack of scarlet fever, which occurred a few weeks before the first fit. In the family history we find the maternal relatives were all "very nervous" and his mother had sick headaches for years. From fifteen to twenty-five years of age she had "fainting with crying and laughing spells"(hysteria). Patient is the youngest in a family of three. The other two children are girls who are very well and unusually bright and intelligent. The remainder of the patient's family and personal history is negative.

CLARKE.—CASE NO. I. C. D.

In March, 1896, patient was admitted to the Craig Colony for treatment. He was found to be very nervous in manner and easily disturbed without apparent cause. He was not capable of sustained attention and was quite noticeably effeminate in all his acts. He had never associated with other boys and because of his infirmity had been under very lax home discipline.

Physical examination: Heart showed mitral murmur and much left ventricular hypertrophy. He often complained of facial flushing and periodic attacks of headaches. The taché cerebralé was observed.

An irregular coarse tremor has always been present, having been much exaggerated when slightly excited. The occipito-frontalis and corrugator supercilli habitually overacted, which action became as marked as a habit spasm when excited. His speech was lisping and stammering; the voice was of a childish tone. A profuse bromic acne was found on face and chest. Tongue was tremulous and bitten equally on both sides. (He had had a series of epileptic attacks three days before admission.) Deep reflexes were all exaggerated and foot clonus was obtainable to a slight extent on both sides. In the absence of other cord symptoms this was not looked upon as being essentially pathological. He weighed 130 lbs., and was 5 feet 6 inches in height. Stigmata: No occipital protuberance, narrow, high-arched palate; left parietal eminence was higher than right. Great excess of uric acid was said to exist in urine before admission, but repeated examinations after admission failed to find it.

Since admission, his attacks have all been hysterical paroxysms by day when awake (except one when he was in a dosing state) and those occurring at night, or when asleep by day, have been typical grand mal epileptic fits, differing in no essential particular from classic epilepsy.

I have personally seen many of his hysterical paroxysms by day. They generally occur in the following manner: After some emotional disturbance of anger or disappointment his face will flush and eyelids close with the well-known shutter-like action seen in hysterics. He then slides to the floor and remains in a rigid state for a few seconds, after which time he keeps his legs and arms in motion, kicking and striking any one who may be within reach. He also makes a great effort to bite and scratch any one touching him. He then rolls over three or four times and a series of convulsive tremors pass over entire body. He will almost always grasp any one's hand, if asked to do so, squeezing much or little as one commands him. Occasionally he opens the eyes wide enough to see that he takes good aim at any one. He rarely injures himself when in the attacks. If a nurse of whom he is fond is standing by him, the attack can be controled by talking to him in an authoritative manner. Although conscious enough to obey simple commands while in this condition, he rarely utters any words or makes any attempt at speech. Respiration is rapid and loud. No stertor, coma or tongue-biting occur in the day attacks. He has but little remembrance of his hysterical acts when he returns to his normal state. No period of depression follows such attacks. These day attacks are designated by his fellow patients as "nervous kicking spells." The contrast between the two kinds of seizures is very marked and has long been a subject of comment by the merest novice of a nurse witnessing both. Frequently patient has had globus hystericus before his day attacks. The paroxysm generally lasts about fifteen or twenty seconds. A rather interesting fact is that bromides have never had any influence over the hysterical paroxysms, but they have frequently postponed the epileptic fits for months.

Patient is gaining much in self-control with consequent improvement in his hysteria, but his epileptic attacks have not materially changed. I have not described them as they are classic tonic and clonic epilepsy. Photograph of Case No. I. is presented herewith.

CASE II.—A. G., female, aged 18, single, no occupation. Admitted to Craig Colony for treatment in May, 1898. Epilepsy began at three. An uncle had epilepsy; aside from that fact family and personal history is negative. No record of hysterical attacks before admission. Physical examination showed a good, well-nourished physique. Heart was irregular and intermittent; pulse-rate, 95; sensibility, normal. Slight paresis of right side remains. Reflexes exaggerated on right side. Weight, 126 lbs.; height, 5 feet, 5½ inches. Her mouth is unusually small and the face evidences a weak mentality. Cause of the epilepsy was attributed to cholera infantum. Aura, epigastric. The real cause of her epilepsy was an infantile cerebral palsy at three, leaving the right side paralytic for four months. Epileptic attacks occur both by night and day, about seven each month.

CLARK.—CASE NO. II. A. G.

Her epileptic attacks are as follows: An attack May 5, 1898; patient had epigastric aura; tonic spasm lasting thirty seconds; body very rigid; hands and feet extended in straight line and lips drawn firmly together. The pupils were dilated to the fullest extent. Violent general clonic spasms, followed tonic period, which lasted about one minute. The thumbs were flexed in palms and knees were widely abducted from the median line of body, and then flexed to the chin. Head was drawn back and to the right side. At the beginning of the clonic period the teeth were forcefully snapped together, biting the tongue severely. She frothed at the mouth and face was purple. She slept after the convulsion in a stertor stage for one half hour. She suffered no depression after. All of her epileptic fits are similar to these.

June 23d patient had a typical paroxysmal hysterical seizure. The eyes were closed and the body was rigid, assuming a strained statue attitude. No response was obtained. She recovered from this paroxysm in a few minutes to pass into the hysterical state, in which condition she remained for eighteen hours. She assumed the passional attitudes described and illustrated so well by Richet. There were zones of anesthesia; pupils were responsive to light and face remained flushed. The fingers were rigid and flexed throughout. Suggestion that stomach-tube should be passed for feeding her, induced her to take food and terminated the hysterical state.

September 14th, following a similar paroxysm with its subsequent phenomena of cataleptic, passional posing, she had a bluish edematous rash over entire body (similar descriptions found in works on hysteria by Sydenham and Charcot). Such eruptions are not infrequent in hysteria, and have since been called the blue and white edema of Charcot and Sydenham. A few hours following this rash, trophic ulcers appeared spontaneously on right arm and right buttock. Neither position was subject to irritation or pressure. Photographs of this patient and her ulcers on buttock are herewith presented. No temperature or other constitutional disturbance appeared with the rash or the ulcers. On several occasions since September 14th she has had similar hysterical paroxysms, followed by erratic and passional posings. During such times she assumes to talk to God, and sings foolish, childish songs. The ulcers heal slowly.

CLARK.—CASE NO. II. SHOWING TROPHIC ULCERS.

In conclusion I would say that Case I. shows but little hysteria in the interparoxysmal state and is probably true hysteria major, alternating with epileptic fits. The patient has hysterical attacks by day and epileptic fits by night.

Case II. shows not only true epilepsy and hysterical paroxysms occurring occasionally, but also many of the interparoxysmal phenomena of hysteria, such as cataleptic and erratic posings and a tropho-neurosis of the skin in characteristic rash and ulcers. Epilepsy and hysteria also exist separately in this case.

Gowers has described a case very similar to this one. His case also was in an infantile hemiplegic, with but slight evidences of paralysis.

## Figures and Tables

**Figure f1:**
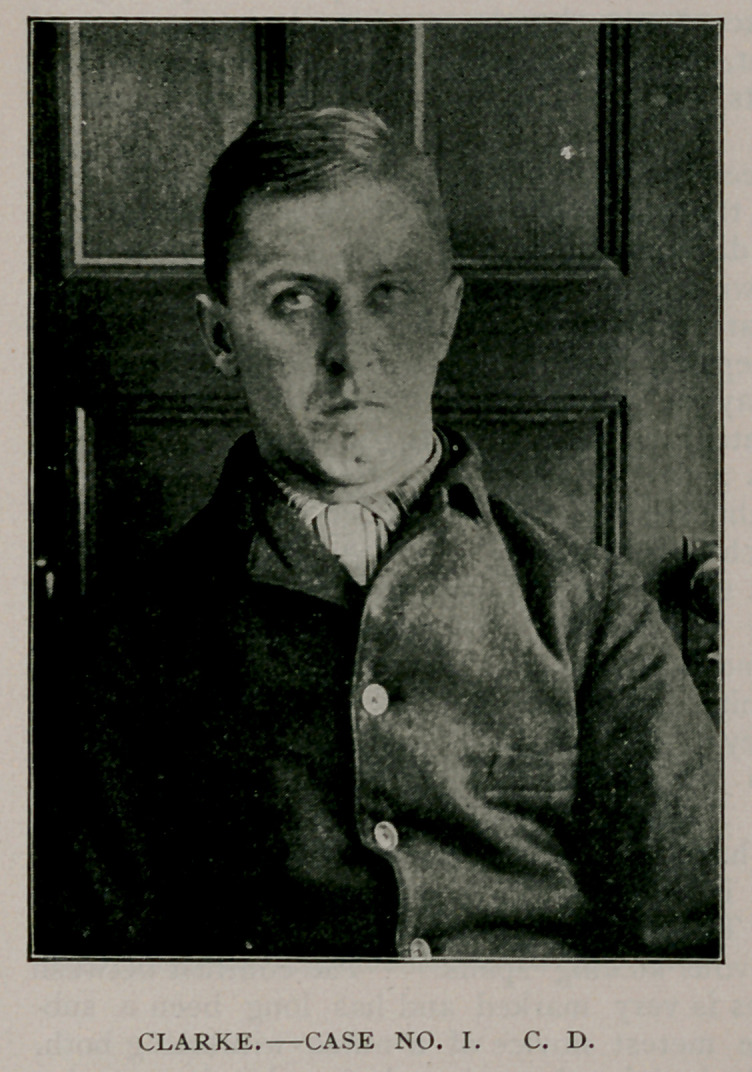


**Figure f2:**
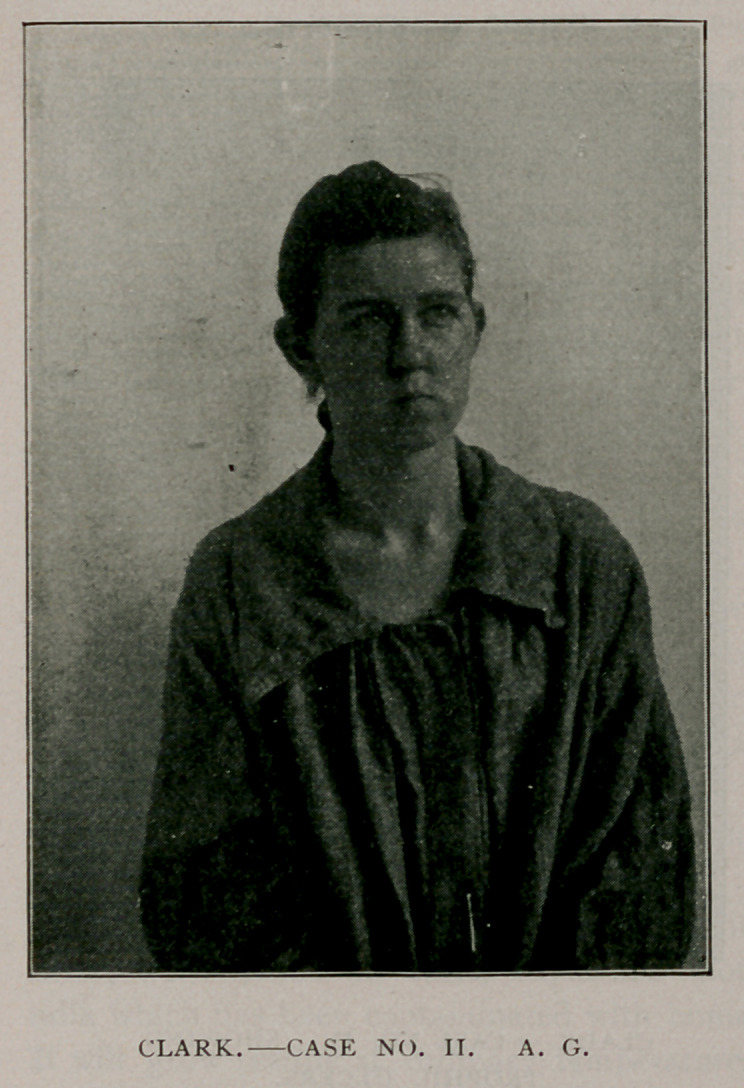


**Figure f3:**